# Apelin, Cortisol, and Doxorubicin-Induced Cardiotoxicity: A Triangle of Actions

**DOI:** 10.3390/cells15131187

**Published:** 2026-06-30

**Authors:** Kinga Dziobiak, Maja Owe-Larsson, Mirosława Chwil, Izabela Róża Janiuk

**Affiliations:** 1 Chair and Department of Experimental and Clinical Physiology, Laboratory of Centre for Preclinical Research, Medical University of Warsaw, Banacha 1b, 02-097 Warsaw, Poland; k.dziobiak@gmail.com; 2 Department of Histology and Embryology, Centre of Biostructure Research, Medical University of Warsaw, Chałubińskiego 5, 02-004 Warsaw, Poland; maja.owe-larsson@wum.edu.pl; 3 Department of Botany and Plant Physiology, University of Life Sciences in Lublin, Akademicka 15, 20-950 Lublin, Poland

**Keywords:** apelin, cortisol, doxorubicin, doxorubicin-induced cardiotoxicity, p-glycoprotein, cardiotoxicity, HPA axis

## Abstract

The mechanisms underlying doxorubicin (DOX) cardiotoxicity include activation of the renin–angiotensin–aldosterone system (RAAS), oxidative stress, mitochondrial dysfunction, calcium overload, and cardiomyocyte apoptosis. Cortisol plays a key role in regulating multiple metabolic, immunological, cardiovascular, and neuroendocrine processes and may additionally influence drug pharmacokinetics by modulating the activity of P-glycoprotein (P-gp). The peptide apelin, through its specific target, angiotensin II protein J receptor (APJ), exerts cardioprotective, antifibrotic, and anti-inflammatory effects. The available data demonstrate that apelin signaling protects against DOX-induced cardiotoxicity, impacts cortisol secretion, and inhibits RAAS. Short-term elevation in cortisol levels, caused by apelin, may reduce inflammation and thus have cardioprotective properties. However, through chronically elevated cortisol levels, apelin may indirectly contribute to peripheral resistance, cardiac remodeling, and myocardial damage, especially when cortisol metabolism by 11β-hydroxysteroid dehydrogenase 2 (11β-HSD2) is altered. This narrative review explores the potential molecular and cellular mechanisms shaping the outcome of apelin–cortisol interplay, offering a potential foundation for developing cardioprotective strategies during anticancer therapy. Future studies should be aimed at assessing the complex interactions between cortisol, apelin, and the RAAS regarding DOX-induced cardiotoxicity.

## 1. Introduction

Doxorubicin (DOX) is one of the most effective and widely used anthracycline antibiotics in anticancer therapy [1]. This drug is used to treat a wide range of cancers, from hematological malignancies, including leukemias and lymphomas, to breast cancer, lung cancer, and sarcomas [2]. DOX acts by intercalating deoxyribonucleic acid (DNA) and inhibiting topoisomerase II, inducing apoptosis and oxidative stress in dividing tumor cells [3]. The main route of DOX metabolism is its enzymatic reduction to doxorubicinol by alcohol dehydrogenase, carbonyl reductase, and aldo–keto reductase [4]. DOX use is associated with numerous side effects, including a significant reduction in white blood cell, red blood cell, and platelet counts [5]; cardiotoxicity [6]; alopecia [7]; nausea and vomiting and oral ulceration [8]; extravasation injury [9]; neurotoxicity and “chemo brain” [10]; hepatotoxicity and nephrotoxicity [11,12]; and adrenal gland damage [13].

DOX-induced cardiotoxicity is one of the most serious adverse effects associated with this drug [14,15]. It is characterized by a progressive decline in left ventricular function that may ultimately lead to irreversible heart failure. The underlying mechanisms are multifactorial and include increased production of reactive oxygen species (ROS), mitochondrial dysfunction, calcium overload, and cardiomyocyte apoptosis [16,17,18]. Additionally, structural alterations such as myocardial fibrosis and microvascular dysfunction further exacerbate cardiac injury [19,20]. When administered to mature cardiomyocytes, DOX forms complexes with topoisomerase IIβ, resulting in double-stranded DNA breaks, impaired transcription, impaired mitochondrial biogenesis, and a secondary increase in ROS [21,22,23]. Other effects include excessive activation of the renin–angiotensin–aldosterone system (RAAS) in the myocardium, increased angiotensin II level, enhanced angiotensin II type 1 receptor (AT_1_) expression, and elevated angiotensin-converting enzyme (ACE) activity, leading to excessive vasoconstriction, oxidative stress, cardiomyocyte apoptosis, and myocardial fibrosis [17,18,24]. Blocking the RAAS with ACE inhibitors or AT_1_ receptor antagonists significantly attenuates these changes, suggesting that excessive RAAS activation is a major pathogenic mechanism of DOX cardiotoxicity [25,26].

Apart from its potential cardiotoxic effects, chemotherapy is a potent systemic stressor that can lead to elevated serum cortisol levels in cancer patients, as demonstrated in observations from clinical therapeutic studies of hormonal responses during treatment [27]. In turn, cortisol may exert both protective and harmful effects on the cardiovascular system, depending on the duration of the patient’s exposure to elevated levels of this hormone. Acute cortisol-induced immunomodulatory mechanisms may promote the resolution of inflammation, the removal of damaged cells, and the transition to the repair phase [28]. However, in situations of chronic stress or metabolic disorders, excessive cortisol secretion can lead to hypertension, insulin resistance, lipid disorders, and cardiovascular damage, e.g., via enhanced RAAS activity and increased expression of β-adrenergic receptors [29,30,31]. As cortisol increases arterial pressure and peripheral resistance, it may increase myocardial susceptibility to anthracycline-induced injury [32]. Simultaneously, DOX may cause adrenal cortex cell damage or indirectly modify cortisol levels by inducing a systemic stress response [6,33,34,35,36]. This is reflected in the results of a prospective study of patients with lymphoma who received the R-CHOP (rituximab, cyclophosphamide, DOX, vincristine, and prednisone) or CHOP (cyclophosphamide, DOX, vincristine, and prednisone) regimen, which includes DOX. Temporary hypothalamic–pituitary–adrenal axis (HPA) suppression and adrenal insufficiency occurred in approximately 30% of patients, with the first cases occurring after the third cycle and the highest incidence after the fifth cycle of chemotherapy [37]. Importantly, HPA axis suppression most often reversed 3–5 weeks after the end of therapy, although in individual cases the impairment persisted longer [37]. These complex interactions emphasize the importance of considering not only DOX cardiotoxicity but also its effects on the HPA axis, as cortisol levels may influence the extent of myocardial injury.

Due to the potentially toxic effects of DOX treatment, there is an urgent need to identify protective mechanisms against tissue damage. An endogenous protective mechanism that counterbalances RAAS overactivation and limits DOX-induced myocardial injury involves the apelinergic system [38]. It is composed of apelin peptides and their G protein-coupled receptor (angiotensin II protein J receptor—APJ). Apelin was isolated from gastric glandular epithelial cells [39]. It is an endogenous peptide, encoded by the *APLN* gene, and the primary product of its translation is a 77-amino acid preproprotein (preapelin). Its enzymatic cleavage produces several apelin isoforms that differ in polypeptide chain length and biological function [40]. Its endogenous forms include apelin-36, apelin-17, apelin-16, and apelin-13, while apelin-12 is an exogenously synthesized form. Their biological activity depends on the peptide length, as long-chain forms become several to several dozen times more active after conversion to short-chain peptides [39]. Apelin isoforms bind to the APJ, which is a rhodopsin-like G protein-coupled receptor (GPCR), identified based on sequence homology with the angiotensin II (Ang II) receptor (AT_1_) [41]. The apelin system (apelin and APJ) acts as an anti-regulatory axis towards the classical Ang II/AT_1_ axis [42]. Though apelin may protect the heart from DOX-induced toxicity [38], on the other hand, DOX may downregulate apelin levels in the plasma and APJ protein expression, as shown in a murine model [43].

The adverse effects of chemotherapy drugs, such as DOX, pose a challenge in modern oncology. This is why new ways to inhibit these mechanisms are being explored. Given that apelin, along with the APJ, may reduce DOX cardiotoxicity, it is essential to clarify the involvement of cortisol signaling in the balance between toxic and protective processes. Therefore, understanding the mechanistic correlations within the cortisol, DOX, and apelin triangle may provide new insights into the mechanisms of myocardial injury and identify potential targets for therapeutic intervention. We hypothesize that, depending on the duration of exposure and local in situ levels, cortisol may either participate in apelin-induced cardioprotective mechanisms or exacerbate the cardiotoxic effects of DOX. Thus, this review aimed to summarize the current knowledge on the interactions between cortisol, DOX, and apelin, with particular emphasis on their role in cardiotoxicity. A comprehensive analysis of original and review publications from the years 1978–2025 was performed. Only papers available in English were included. The following keywords were searched in the PubMed database: “cortisol” AND “doxorubicin” OR “doxorubicin-induced cardiotoxicity” OR “p-glycoprotein” OR “HPA axis”, “apelin” AND “doxorubicin” OR “cortisol”.

## 2. Cortisol

Cortisol is synthesized from cholesterol in the adrenal cortex, primarily in spongiocytes of the zona fasciculata [44,45,46,47]. The first rate-limiting step in the entire cortisol synthesis process is the transport of cholesterol to the inner mitochondrial membrane by the steroidogenic acute regulatory protein (StAR) [48]. In the mitochondria, the enzyme cytochrome P450scc (desmolase/CYP11A1) converts cholesterol into pregnenolone, initiating the steroidogenic pathway [49,50]. Pregnenolone is subsequently converted in the endoplasmic reticulum into 17(OH)-pregnenolone, 17(OH)-progesterone, and 11-deoxycortisol, which is transformed to cortisol in the mitochondria; cortisol, in turn, is secreted into the systemic circulation [51].

Cortisol release follows the circadian rhythm, and thus, it is regulated by the suprachiasmatic nucleus, which activates the HPA axis [52,53]. After receiving a stressful stimulus, the hypothalamus releases corticotropin-releasing hormone (CRH), which stimulates the anterior pituitary gland. It results in the production of the adrenocorticotropic hormone (ACTH), which stimulates the adrenal cortex to synthesize and release cortisol [54]. Following its synthesis from circulating cholesterol, approximately 95% of cortisol is transported in the bloodstream bound to α2-globulin, known as transcortin or corticosteroid-binding globulin (CBG). The limited binding capacity of CBG in combination with excessive cortisol production leads to an accumulation of free cortisol in the plasma [55].

When the glucocorticoid receptor (GR) is inactive, it is primarily located in the cell cytoplasm. It is part of a large multiprotein complex that consists of the receptor polypeptide, several other proteins, and two molecules of heat shock protein 90 (hsp90) [56]. The receptor dissociates from hsp90 upon ligand binding and then translocates to the nucleus [57]. GRs are expressed widely, with major target tissues including the liver, skeletal muscle, bone tissue, adipose tissue, connective tissue, pancreas, urinary system, intestinal system, central nervous system, immune system, and cardiovascular system, where cortisol exerts its physiological effects. Conversely, chronic or recurrent high cortisol concentrations can lead to decreased sensitivity of GRs, their desensitization, and impaired signal transduction. This results in, e.g., attenuation of the hormone’s classic anti-inflammatory effects despite its high blood levels [58]. Therefore, the effects of cortisol on various targets, including the immune and vascular systems, should be interpreted in the context of its duration of action. Short-term and chronic effects may have different effects on repair, inflammatory, and neurohormonal processes in the body, such as cardiotoxicity [59].

Cortisol exerts an anti-inflammatory effect through multiple mechanisms [60,61]. It increases the expression of anti-inflammatory factors, such as interleukin 10 (IL-10) and annexin A1 (lipocortin-1), a cytosolic phospholipase A2 inhibitor [62,63]. Cortisol induces apoptosis of T helper 1 (Th1) cells and cytotoxic cluster of differentiation 8 (CD8^+^) T lymphocytes [64]. The expression of interleukin 2 (IL-2) and its receptor on B lymphocytes is reduced by glucocorticoids (GCs) [65], resulting in the inhibition of their proliferation and differentiation into plasma cells and reduced immunoglobulin production [66]. Cortisol reduces the number of monocytes and macrophages and their ability to produce cytokines and present antigens, but it increases the phagocytosis of apoptotic cells by macrophages [58,67]. This hormone also increases the neutrophil count by promoting their demarginalization and release from the bone marrow; however, such neutrophils are less active [67,68,69].

Following chemotherapy-induced myocardial injury, particularly in the context of anthracycline-related cardiotoxicity, the inflammatory response fulfills an adaptive function. However, its effective suppression is essential for proper cardiac regeneration and limiting secondary myocardial damage. In the acute phase of cardiotoxicity, some cortisol-induced immunomodulatory mechanisms may promote controlled resolution of inflammation, facilitate the removal of damaged cardiomyocytes, and support the transition from the inflammatory to the repair phase [28]. Cortisol increases the expression of the anti-inflammatory cytokine IL-10, which inhibits the activity of transcription factors NF-κB and AP-1, leading to reduced expression of proinflammatory cytokines and inflammatory mediators [60,61,70]. Another process that promotes the reduction of local inflammation is the expression of annexin A1 (lipocortin-1). It acts as a pro-resolution mediator, limiting the migration and activation of leukocytes and supporting the suppression of the inflammatory response [71]. Finally, cortisol increases the ability of macrophages to phagocytose apoptotic cells (efferocytosis) [72]. The above mechanisms indicate that in the acute phase, cortisol may promote controlled resolution of inflammation and create conditions enabling effective regeneration of heart muscle following anthracycline-induced cardiotoxicity, particularly DOX-induced myocardial injury.

Unfortunately, long-term activation of the GR in immune cells may disrupt the coordination of the inflammatory and repair response, which in tissues with limited regenerative potential, such as the heart muscle, may result in abnormal healing and unfavorable remodeling [59]. Long-term exposure to glucocorticoids leads to persistent inhibition of the IL-2/IL-2R axis, which results in reduced lymphocyte proliferation and function [65], as well as impaired ability of macrophages to precisely regulate the inflammatory response, i.e., to produce signaling cytokines and communicate with other cells of the immune system [58,73].

In the cardiovascular system, cortisol increases the expression of β-adrenergic receptors, increasing arterial pressure and peripheral resistance [31]. This mechanism may cause increased susceptibility to myocardial injury induced by anthracycline drugs [32]. Moreover, this hormone inhibits the expression of the *NOS3* gene, leading to a reduction in endothelial nitric oxide synthase (eNOS) messenger ribonucleic acid (mRNA) and protein levels in endothelial cells, and, consequently, decreased vasodilation, increased peripheral resistance, and higher blood pressure [74]. Suppression of NOS3/eNOS by cortisol may enhance vascular dysfunction during anthracycline therapy [75]. Cortisol also acts locally in vessels by modulating the enzymes 11β-hydroxysteroid dehydrogenase 1 (11β-HSD1) and 11β-hydroxysteroid dehydrogenase 2 (11β-HSD2) [76]. 11β-HSD1 acts as a reductase, converting inactive cortisone into active cortisol [77], while 11β-HSD2 inactivates cortisol to cortisone [78]. Under physiological conditions, vascular structures express both 11β-HSD1 and 11β-HSD2, which allows for local regulation of active cortisol levels—either by activating it via HSD1 or by inactivating it via HSD2. Although cortisol itself does not directly reduce 11β-HSD2 expression, chronic exposure to high cortisol concentrations can lead to enzyme overload, resulting in its relative deficiency, particularly in the kidneys and endothelium [76,79]. Importantly, disturbances in local cortisol metabolism, e.g., anthracycline treatment, could potentiate cardiac susceptibility to injury [80,81].

Cortisol has a similar affinity to mineralocorticoid receptors (MRs) as aldosterone. However, under physiological conditions, it is inactivated by 11β-HSD2, protecting the receptor from activation by glucocorticoids [82,83]. In endothelial damage or kidney disease, i.e., when the activity or expression of 11β-HSD2 is reduced, cortisol can agonistically activate MR, which results in increased sodium and water retention and increased blood pressure [84,85]. Cortisol also increases angiotensinogen gene expression in the liver and other tissues. This occurs especially during the perinatal period and in metabolic stress [26]. Angiotensin is a member of the RAAS, the primary hormonal axis controlling intravascular volume and blood pressure [25]. Reduced renal perfusion in the afferent arteriole, low NaCl concentration in the macula densa, and renal activation of sympathetic β_1_-adrenergic receptors stimulate renin release from juxtaglomerular cells [86]. Renin cleaves liver-derived angiotensinogen (AGT) to angiotensin I, which is converted by ACE to angiotensin II (Ang II). Ang II, acting on AT_1_ receptors, induces potent vasoconstriction; stimulates aldosterone synthesis in the glomerular layer; increases tubular sodium reabsorption (directly and indirectly, via aldosterone); and promotes antidiuretic hormone (ADH) release, thirst, oxidative stress, inflammation, and fibrosis [87,88]. AT_2_ receptor signaling provides counterregulation. The complementary, “non-classical” axis, ACE2/Ang-(1–7)/Mas, exerts vasodilatory, natriuretic, and antifibrotic effects and balances AT_1_-mediated effects [89]. In summary, cortisol stimulates and enhances RAA signaling, which results in increased sodium and water retention and increases blood pressure [90]. Chronic RAAS activation increases myocardial stiffness, fibrosis, and diastolic dysfunction [91].

The mitogen-activated protein kinase (MAPK) pathway, which encompasses several major subpathways, such as extracellular signal-regulated kinases (ERK1/2), c-Jun N-terminal kinases (JNK), and p38 MAPK, can also be modified by cortisol [92]. Under physiological conditions, the MAPK pathway regulates cell proliferation and differentiation, participates in cell cycle control, and participates in the immune response [93]. In the heart, MAPK plays a crucial role in regulating cardiomyocyte function and adaptation to hemodynamic stress [94,95]. The MAPK pathway becomes potentially harmful when its activation is excessive, chronic, or inappropriate to the stimulus. Under such conditions, it leads to increased oxidative stress, activation of inflammatory processes, induction of cardiomyocyte apoptosis, and myocardial remodeling [96]. This means that chronic MAPK activation may be involved in the development of cardiotoxicity, including that induced by anthracyclines such as DOX [97].

## 3. DOX and Cortisol Levels

Drug-induced changes in cortisol levels can result from cytotoxic effects towards adrenal cortex cells [36], inhibition of steroidogenic enzymes [98], and modulation of CRH/ACTH secretion [99]. DOX represents a well-documented example of a drug that directly impairs adrenal function due to destruction of adrenal cells [34]. Probable causes include the generation of ROS through redox conversion of its quinone group, which leads to damage to membrane lipids and mitochondrial DNA and proteins, as well as inhibition of topoisomerase II, which leads to chromosomal damage and cell cycle arrest [6]. At high concentrations, DOX directly disrupts the cell membrane and impairs the exocytosis mechanism [13]. However, it was also suggested that the toxic damage to the adrenal glands that causes the lowering of cortisol is mediated at the pituitary or hypothalamic level [33].

Paradoxically, an increase in cortisol levels after a one-hour intravenous infusion of DOX (12.5 mg/kg) was observed in a rabbit model [100]. These changes were not attributed to a direct effect on the adrenal cortex but were considered a response to the metabolic and hemodynamic stress induced by the drug [100]. A study in an ex vivo model using perfused bovine adrenal glands revealed degenerative changes in the adrenal cortex, such as atrophic cells, cytoplasmic shrinkage, and condensed nuclei in tissues exposed to a P-glycoprotein (P-gp) inhibitor in combination with DOX or mitotane [34]. After P-gp blockade, the cytotoxic effect of these drugs is revealed by a significant decrease in cortisol secretion, suggesting that DOX-induced damage to the adrenal cortex may be partially masked by the action of the efflux transporter [34]. In another study, the incidence of transient adrenal insufficiency in patients with diffuse large B-cell lymphoma was assessed after five cycles of R-CHOP chemotherapy, which includes DOX administration along with other chemotherapeutic agents [37]. At the time of diagnosis, baseline morning serum cortisol levels in all patients were within the normal range, with a mean level of 11.1 μg/dL (S.D., 3.0 μg/dL). After the fifth cycle of therapy, 20% of patients were diagnosed with adrenal insufficiency, indicating that chemotherapy, including DOX, may exert a damaging effect on the adrenal cortex [37].

On the other hand, the available literature does not provide clinical studies directly documenting the relationship between cortisol levels and changes in DOX metabolism. However, there is strong mechanistic evidence indicating that activation of the HPA axis may influence the biotransformation of DOX and its functional elimination via membrane transporters [101,102]. The dominant biotransformation pathway of DOX is the reduction of the carbonyl group to doxorubicinol (DOXOL), a metabolite with less anticancer activity but important in the context of anthracycline toxicity, especially cardiotoxicity. This reduction occurs primarily via cytosolic NADPH-dependent carbonyl reductases and aldo–keto reductases in the liver and other tissues [102]. A particularly important finding is the demonstration that 11β-HSD1 (an enzyme classically associated with local glucocorticoid regulation) participates in the metabolism of DOX to DOXOL and may be responsible for a significant portion of DOXOL formation in human hepatocytes [101]. DOX exposure is also influenced by membrane transporters, especially P-gp, which limit intracellular concentrations of many xenobiotics through active efflux, which translates into distribution and functional elimination [103]. 11β-HSD1 is considered a key regulator of tissue availability of active glucocorticoids, and its expression and activity are subject to hormonal regulation and conditions associated with glucocorticoid excess [79,101,104]. It has been shown that activation of the GR by dexamethasone can increase the expression and activity of 11β-HSD1 [105,106]. Cytosolic NADPH-dependent carbonyl reductases are involved in the reduction of DOX to DOXOL, but at the same time, they are involved in the metabolism of glucocorticoids by reducing cortisol to 20β-dihydrocortisol [107]. Experimental data show that glucocorticoids can increase the expression and activity of efflux transporters, including P-gp, and the effect is partially dependent on the GR. DOX is a substrate of transport processes; increased P-gp activity may lower intracellular exposure in selected tissues and modulate drug distribution/resistance [103]. Studies on the interplay between doxorubicin (DOX) and glucocorticoid pathways—glucocorticoid-associated drug metabolism, cortisol regulation, and adrenal function—are summarized in Table 1.

## 4. Cortisol and DOX-Induced Cardiotoxicity

Cortisol may enhance cardiotoxicity by various mechanisms [32,75,80,81]. This hormone may influence DOX-induced cardiac damage not only as a standalone toxic agent but also as a modulator of the environment [80]. It may alter the susceptibility of cardiomyocytes, endothelium, and fibroblasts to DOX via pathways that are partially shared with the mechanisms of anthracycline cardiotoxicity. Key intersections include oxidative stress [108], endothelial dysfunction [74], MR/RAAS signaling [26], impaired post-inflammatory repair, fibrosis, and the cardiomyocyte response to DNA damage and mitochondrial stress.

**Figure 1:**
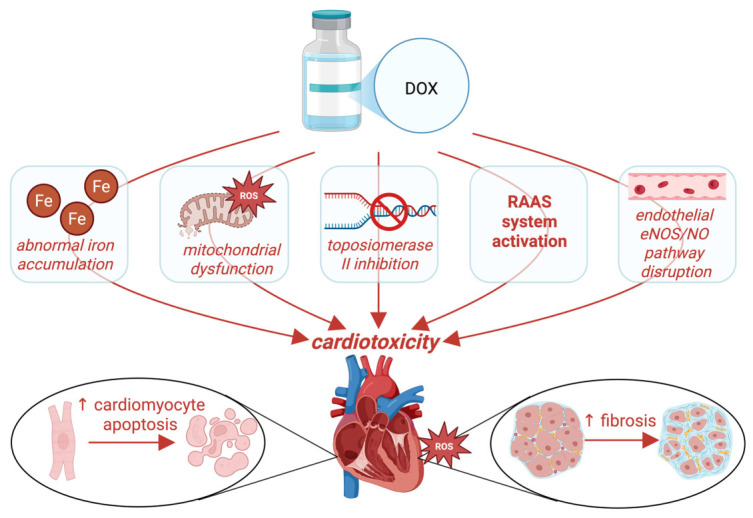
The impact of DOX on the cardiovascular system. DOX—doxorubicin, eNOS—endothelial nitric oxide synthase, NO—nitric oxide, RAAS—renin–angiotensin–aldosterone system, ROS—reactive oxygen species. Created in BioRender. Owe-Larsson, M. (2026) https://BioRender.com/u44tj4r.

Doxorubicin induces cardiotoxicity through a complex set of interrelated molecular, cellular, and tissue mechanisms. All of these processes lead to fibrosis, increased myocardial stiffness, adverse remodeling, and a gradual deterioration of left ventricular systolic and diastolic function. Clinically, this process can manifest as anthracycline-induced cardiomyopathy and, in advanced cases, heart failure [91]. A summary of the cardiovascular effects of DOX is presented in Figure 1.

One of the primary cardiotoxicity-promoting mechanisms is the interaction of DOX with topoisomerase IIβ in mature cardiomyocytes [22]. Formation of the DOX–topoisomerase Iiβ-DNA complex leads to double-stranded DNA breaks, impaired transcription, and impaired mitochondrial biogenesis. Consequently, there is a secondary increase in reactive oxygen species production, energy deficit, and activation of cellular damage pathways [21]. The second central element of DOX cardiotoxicity is mitochondrial dysfunction. Cardiomyocytes are particularly susceptible to this type of damage because their proper function depends on constant ATP production [6]. DOX can accumulate in mitochondria, disrupt the respiratory chain, and damage mitochondrial DNA and mitochondrial membranes. This leads to decreased ATP production, loss of mitochondrial membrane potential, impaired calcium homeostasis, and further increased ROS production. This creates a cycle in which damaged mitochondria become both the target and the source of escalating oxidative stress [109]. DOX can also promote abnormal iron accumulation, particularly in mitochondria, and the presence of unbound or improperly stored iron enhances free radical reactions. This leads to lipid peroxidation, damage to cell and mitochondrial membranes, and increased susceptibility of cardiomyocytes to ferroptosis, an iron-dependent form of regulated cell death [110,111]. Moreover, damage to DNA, mitochondria, and membrane lipids, as well as disruption of calcium homeostasis, leads to the activation of various forms of regulated cell death, including necroptosis, pyroptosis, and impaired autophagy and mitophagy [6]. The best-described of these is mitochondrial apoptosis, which involves an imbalance between pro- and antiapoptotic proteins, the release of cytochrome c, and the activation of caspases [96,110,112]. The mechanism that links many of these processes is oxidative stress. ROS damage proteins, lipids, and DNA; exacerbate mitochondrial dysfunction; activate inflammatory and proapoptotic pathways; and promote fibrosis [113,114]. In response to cardiomyocyte death and stress, damage signals are released, and an inflammatory response is activated. While short-term inflammation may assist in the removal of damaged cells, its persistence promotes the production of proinflammatory cytokines, immune cell recruitment, fibroblast activation, and extracellular matrix remodeling [115].

Consistently, a key mechanism by which cortisol may promote myocardial remodeling is the activation of the MR in an environment of oxidative stress [108]. Under physiological conditions, cortisol has a high affinity for MR, but its access to this receptor is limited by 11β-HSD2, which inactivates cortisol to cortisone and thus protects the MR from excessive activation by glucocorticoids. When this protection is insufficient or when the cellular redox state is altered, cortisol can act as a functional agonist for MR [116]. Activation of MR in cardiovascular tissues triggers redox-sensitive pathways, including increased activity of NADPH oxidases, leading to further ROS production [117]. This creates a positive feedback loop: oxidative stress promotes pathological activation of MR by glucocorticoids, and activated MR increases ROS production. Concurrently, MR signaling promotes the expression of proinflammatory and profibrotic mediators, fibroblast activation, and collagen deposition in the extracellular matrix. These processes result in fibrosis, increased myocardial stiffness, diastolic dysfunction, and adverse remodeling [118]. Chronic glucocorticoid exposure may create a pro-oxidative environment that lowers the threshold for DOX-induced myocardial injury [119].

DOX also affects the endothelium and microcirculation. Damage to endothelial cells is associated with an increase in oxidative stress, dysfunction of endothelial mitochondria, and disruption of the eNOS/NO pathway [120]. The decrease in nitric oxide bioavailability leads to impaired vasodilation, disturbances of vascular homeostasis, and deterioration of myocardial perfusion [75,120,121]. As cortisol inhibits the expression of the *NOS3* gene, it decreases the amount of the eNOS protein in endothelial cells. These metabolic changes result in reduced vasodilation, increased peripheral resistance, and higher blood pressure [74].

As mentioned earlier, DOX causes excessive activation of the RAAS in the myocardium, increased angiotensin II levels, increased expression of the AT_1_ receptor, and increased ACE activity. Further consequences of this process include excessive vasoconstriction, oxidative stress, cardiomyocyte apoptosis, and myocardial fibrosis. Attenuation of these changes has been observed after blocking the RAAS with ACE inhibitors or antagonists [25,26]. Cortisol also affects the RAAS by increasing angiotensinogen gene expression in the liver and other tissues [26]. It causes increased sodium and water retention and elevates blood pressure [90]. Chronic activation of the RAAS increases myocardial stiffness, fibrosis, and diastolic dysfunction [91].

Moreover, in experimental models of DOX-induced cardiotoxicity, β-adrenergic signaling shows subtype-specific responses. β1-adrenergic activation was associated with a more deleterious response, whereas β2-adrenergic signaling may exert protective effects. Therefore, cortisol-driven enhancement of the β-adrenergic response may increase susceptibility to anthracycline-induced myocardial injury, depending on the balance of β-adrenergic receptor subtype signaling [32]. Modulation of the MAPK pathway by cortisol could also be involved in exacerbating cardiotoxicity [92,97].

Following cardiomyocyte injury, the inflammatory response is necessary to remove dead cells and initiate tissue repair, but it must be properly resolved to avoid progression to chronic inflammation and fibrosis [122]. Short-term activation of the glucocorticoid receptor can exert anti-inflammatory and pro-resolution effects, including by inhibiting the transcription factors NF-κB and AP-1, reducing the expression of proinflammatory cytokines, and inducing mediators of inflammation resolution, such as annexin A1 [123]. Glucocorticoids also influence the phenotype of macrophages, promoting their transition toward cells involved in inflammation resolution, efferocytosis, and tissue repair [124]. In this sense, an acute increase in cortisol after injury may limit secondary inflammatory damage. However, chronic or excessive exposure to glucocorticoids may disrupt the normal repair sequence. Excessive suppression of the early inflammatory response may limit myeloid cell/macrophage recruitment, impair the removal of damaged cells, and attenuate signals required for angiogenesis and tissue regeneration [125]. In a zebrafish model of cardiac regeneration, glucocorticoid treatment inhibited the early immune response following cardiac injury, reduced phagocytic cell recruitment, and subsequently impaired angiogenesis and cardiomyocyte proliferation during repair [125].

Studies investigating glucocorticoid-related mechanisms and pathways relevant to DOX-induced cardiotoxicity are presented in Table 2.

**Figure 2:**
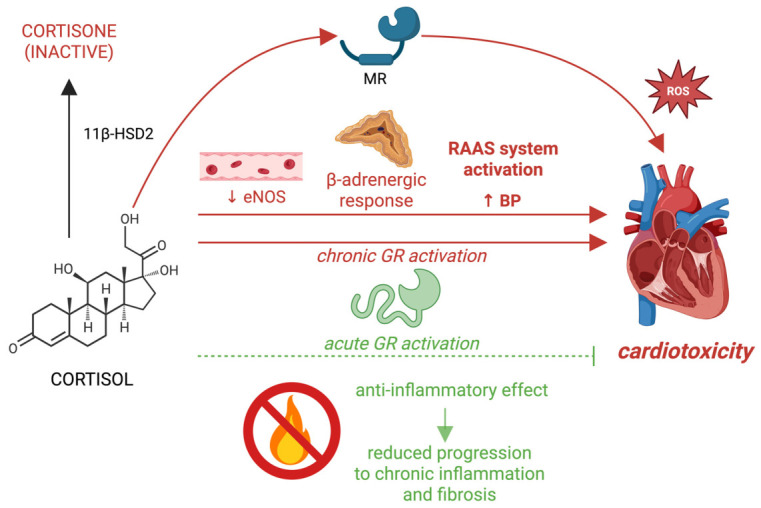
Cardiac effects of cortisol. Red arrows indicate the cardiotoxic effects of cortisol, green arrows indicate its cardioprotective effects, and the black arrow indicates the enzymatic inactivation of cortisol. 11β-HSD2—11β-hydroxysteroid dehydrogenase 2; BP—blood pressure; eNOS—endothelial nitric oxide synthase; GR—glucocorticoid receptor; MR—mineralocorticoid receptor; RAAS—renin–angiotensin–aldosterone system; ROS—reactive oxygen species. Created in BioRender. Owe-Larsson, M. (2026) https://BioRender.com/l5g0e6p.

Taken together, cortisol may modulate DOX-induced cardiotoxicity at vascular, oxidative, neurohormonal, inflammatory, and fibrotic levels. By converging with DOX on endothelial dysfunction, ROS generation, MR/RAAS-dependent remodeling, and dysregulated inflammatory repair, cortisol may lower the threshold at which anthracycline-induced molecular injury progresses into structural myocardial damage. Disturbances in local cortisol metabolism may contribute to enhanced susceptibility of the heart to injury [80,81]. Therefore, the role of cortisol should be interpreted as context-dependent: acute glucocorticoid signaling may support anti-inflammatory resolution, whereas chronic exposure or MR-dominant signaling may favor fibrosis, adverse remodeling, and progressive cardiac dysfunction. A summary of the effects exerted by cortisol on cardiotoxicity is presented in Figure 2.

Given that chemotherapeutic agents—particularly anthracyclines—exhibit cardiotoxic properties and engage in complex metabolic interactions with cortisol, novel therapeutic strategies are urgently needed to mitigate these adverse effects. The apelinergic system emerges as a promising candidate due to its potential to protect against cardiac toxicity.

## 5. The Apelinergic System

Apelin is one of the best-known endogenous regulators of cardiovascular function. It exerts a strong positive inotropic effect, ranking among the most potent natural substances increasing the force of myocardial contraction [126,127]. The underlying mechanism involves apelin’s capacity to elevate intracellular Ca^2+^ concentration in cardiomyocytes [128,129]. Simultaneously, apelin exerts a hypotensive effect, favorably influencing systemic hemodynamics [130,131,132].

At the molecular level, apelin acts as an antagonistic regulator of the classical RAAS axis [133,134]. By activating the APJ, apelin inhibits the activation of the Ang II/AT_1_R axis and counteracts the adverse effects of Ang II, such as vasoconstriction, oxidative stress, inflammation, and myocardial fibrosis, by activating ACE2 and enhancing the Ang-(1–7) pathway [133,135,136,137]. Apelin increases ACE2 expression and enhances the conversion of Ang II to angiotensin-(1–7), which exhibits vasodilatory and anti-inflammatory effects by interacting with the Mas receptor [42,135]. Additionally, due to the ability to form heterodimers with the AT_1_ receptor, the apelin system directly attenuates Ang II/AT_1_ signaling [137]. As a result, apelin creates a physiological counterbalance to the RAAS, demonstrating marked cardioprotective properties—reducing oxidative stress, inhibiting myocardial remodeling and fibrosis, and improving systolic and diastolic function [133,134]. Due to these properties, apelin is considered one of the key endogenous factors protecting the heart against damage caused by excessive activation of the RAAS and oxidative stress, including in the course of heart failure and drug-induced cardiotoxicity [137,138].

The translational relevance of apelin-based cardioprotection, however, may be limited due to apelin’s potential to cause both vasodilation and vasoconstriction, depending on the vascular bed [139]. Moreover, apelin and its isoforms have biological half-lives; their intracellular signaling pathways are highly complex, and more than one APJ receptor subtype may exist [139].

## 6. The Influence of Apelin on the Interplay Between DOX and Cortisol

The available data demonstrate that apelin signaling protects against DOX-induced cardiotoxicity. In mice lacking the APJ receptor and exposed to DOX, profound cardiac contractile dysfunction, increased mortality, increased protein damage, and impaired autophagy were observed. In contrast, APJ overexpression or apelin administration protected against DOX-induced cell death, as shown in cell lines [43]. Apelin also improves electromyographic and echocardiographic function. In rats, apelin-13 prevented the DOX-induced QT and QTc interval prolongation and improved left ventricular systolic parameters [140]. Another mechanism protecting against DOX-induced damage involves the activation of APJ signaling pathways. This stimulates the intracellular PI3K/Akt and ERK1/2 signaling cascades, which upregulate autophagy via transcription factor EB (TFEB). Consequently, regenerative mechanisms are enhanced, accompanied by a reduction in oxidative stress and DNA damage, through APJ activation [43]. However, considering that the apelin pathway is highly activated in treatment-resistant chondrosarcoma cells, it may potentially exert a harmful role, mediating drug resistance [141]. Consequently, the apelin system may play a dual role, offering cardioprotection against DOX toxicity but simultaneously contributing to chemoresistance in neoplastic cells.

Biological effects of apelin are pleiotropic and involve modulation of the immune response, reduced release of pro-inflammatory cytokines, improved insulin sensitivity, and activation of AMP-activated protein kinase (AMPK) [142]. Importantly, apelin is also involved in the regulation of the HPA axis. In the hypothalamus, upon binding to APJ in the paraventricular nucleus (PVN), apelin stimulates the release of CRH and vasopressin (AVP) [143,144]. These neuropeptides synergistically activate the anterior pituitary to secrete ACTH, which in turn stimulates the adrenal cortex to synthesize and release cortisol [145,146]. Through this mechanism, apelin contributes to the neuroendocrine response to stress and may serve as a modulator of glucocorticoid secretion, integrating cardiovascular, metabolic, and stress-related pathways [143,147,148].

Apelin-induced AVP and CRH release have been demonstrated in vitro and in vivo, showing a strong positive correlation between apelin and CRH levels [149,150]. Increased levels of APJ mRNA were found after stress or adrenalectomy [151]. Apelin-stimulated basal ACTH secretion in rats was partially blocked by pretreatment with a CRH antagonist [152,153]. The APJ is located in the adrenal cortex, suggesting the possibility of an autocrine/paracrine action of apelin in the production of glucocorticoids [154,155]. pGlu-apelin-13, the pyroglutamyl form of apelin-13, increases ACTH and corticosterone levels [143]. Overall, the above data indicate that apelin represents one of the mechanisms that may increase cortisol levels and impact the DOX-induced effects.

Cortisol affects P-gp, an efflux pump responsible for removing drugs and toxins from cells, which is important in drug pharmacokinetics and cancer multidrug resistance [156,157]. P-gp is a member of the ATP-binding cassette 1 subfamily B (ABCB1). It uses energy from ATP hydrolysis to actively export endogenous and exogenous substrates into the periphery [156]. Cortisol is both a substrate and an inducer of P-gp. Its presence in the cellular environment increases P-gp expression in cancer cells. P-gp expression is regulated by transcriptional mechanisms under the influence of steroids, indicating that steroid hormones can induce expression of the multidrug resistance 1 (*MDR1*/*ABCB1*) gene encoding P-gp [157]. This may mitigate the negative side effects of DOX. Moreover, as cortisol exerts an immunosuppressive effect, the increase in its levels caused by apelin may be beneficial against DOX-induced cardiotoxicity. Indeed, immunosuppressing agents have been previously shown to exert cardioprotective effects during DOX treatment [158].

**Figure 3:**
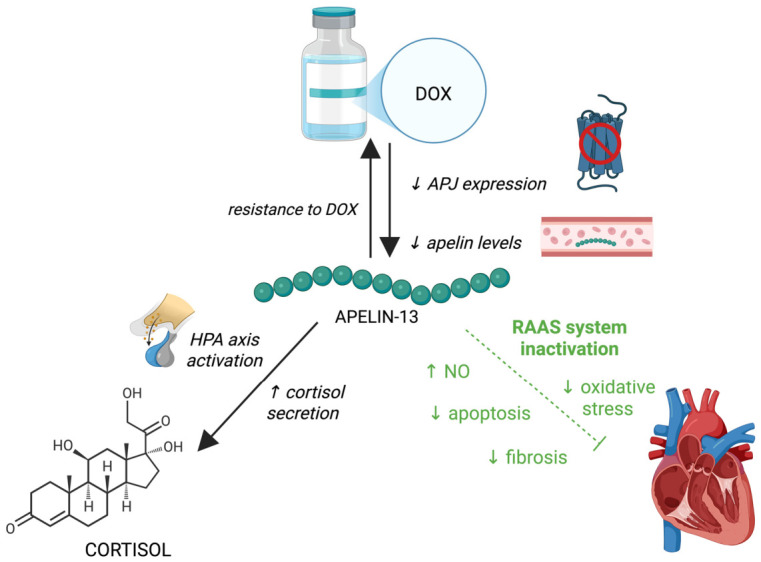
The interplay between apelin-13, cortisol, doxorubicin, and the heart. APJ—apelin receptor; DOX—doxorubicin; HPA—hypothalamic–pituitary–adrenal axis; NO—nitric oxide; RAAS—renin–angiotensin–aldosterone system. The green line indicates the cardioprotective effects of apelin-13. Black arrows indicate the direction of influence among the factors. Created in BioRender. Owe-Larsson, M. (2026) https://BioRender.com/pxth08v.

Nevertheless, enhanced P-gp activity may have a negative influence on the treatment of the underlying cancer disease. Furthermore, the action of the apelinergic system towards RAAS in the DOX-induced cardiotoxicity paradigm is not fully clarified. As cortisol increases the activity of the RAAS [26] and induces oxidative stress [108] and endothelial dysfunction [74], the apelin-induced rise in cortisol may indirectly contribute to peripheral resistance, cardiac remodeling, and myocardial damage. On the other hand, it is well established that the apelin system directly inhibits the activity of RAAS. The role of apelin in the interplay between DOX and cortisol, as well as its cardioprotective properties, is demonstrated in Figure 3.

The available studies on apelin/APJ signaling, HPA-axis activation, and cortisol-related mechanisms relevant to DOX-induced cardiotoxicity are described in Table 3. Future studies should be aimed at assessing the complex interactions between cortisol, apelin, and the RAAS regarding DOX-induced cardiotoxicity.

## 7. Conclusions

The relationship between DOX, cortisol, and apelin is complex and multidirectional, particularly in terms of their influence on the heart and their cardiotoxic/cardioprotective potential.

DOX exhibits potent cardiotoxic properties. A key mechanism is excessive activation of the RAAS, leading to increased expression of AT_1_ receptors, increased oxidative stress, fibrosis, and cardiomyocyte apoptosis. The RAAS is, in part, activated by cortisol, initially suggesting its possible harmful effect in DOX-induced cardiotoxicity. Moreover, cortisol itself may exert negative effects on the cardiovascular system, especially in the case of chronic exposure to this hormone. Cortisol may exacerbate endothelial dysfunction, ROS generation, MR/RAAS-dependent remodeling, and dysregulated inflammatory repair, lowering the threshold at which DOX-induced cardiotoxicity advances into structural damage.

However, glucocorticoids, including cortisol, stimulate the expression of P-gp, ABCB1, which acts as an efflux transporter that removes xenobiotics, including DOX, from target cells. In cardiomyocytes, this mechanism may limit intracellular drug accumulation and reduce its toxicity. Cortisol’s anti-inflammatory properties may also be valuable in attenuating DOX-induced cardiotoxicity. On the other hand, DOX itself may cause adrenal damage and thus lead to decreased cortisol secretion.

Apelin, particularly in the form of pGlu-apelin-13, plays a dual role in this system. Firstly, it exhibits direct cardioprotective effects associated with inhibiting the adverse effects of RAAS activation. Apelin increases ACE2 expression and enhances the ACE2/Ang-(1–7) axis, thereby antagonizing the classical Ang II/AT_1_ pathway. Furthermore, activation of APJ leads to increased nitric oxide production, activation of the PI3K/Akt and AMPK pathways, and, consequently, to reduced oxidative stress, apoptosis, and fibrosis in the myocardium. However, DOX decreases plasma apelin levels and APJ receptor expression. Secondly, apelin stimulates the HPA axis, promoting cortisol secretion, which may influence the resistance to DOX via P-gp. The influence of cortisol on apelin levels and activity is unknown.

In summary, apelin may reduce DOX cardiotoxicity both directly, by modulating the RAAS and activating cardioprotective signaling pathways, and indirectly, via acute cortisol exposure-related and P-gp-dependent mechanisms. However, the latter mechanism still requires direct evidence linking apelin-induced cortisol changes to altered cardiotoxicity outcomes. The cardiovascular impact of cortisol is, on the other hand, twofold: acute GR activation facilitates anti-inflammatory resolution, but chronic exposure or MR overactivation may trigger myocardial fibrosis, adverse remodeling, and progressive heart failure. Further studies are essential to elucidate the complex relationship among cortisol, apelin, and the RAAS, as well as their impact on DOX-induced cardiotoxicity.

## Figures and Tables

**Table 1:** Summary of key studies addressing the interplay between doxorubicin (DOX) and glucocorticoid pathways: glucocorticoid-associated drug metabolism, cortisol regulation, and adrenal function. 11β-HSD1—11β-hydroxysteroid dehydrogenase type 1; ACTH—adrenocorticotropic hormone; AKR—aldo–keto reductase; BCRP—breast cancer resistance protein; CBR1—carbonyl reductase 1; C/EBPβ—CCAAT/enhancer-binding protein beta; C/EBPδ—CCAAT/enhancer-binding protein delta; CHOP—cyclophosphamide, doxorubicin, vincristine, and prednisone; DLBCL—diffuse large B-cell lymphoma; DOX—doxorubicin; DOXOL—doxorubicinol; GR—glucocorticoid receptor; MRP2—multidrug resistance-associated protein 2; P-gp—P-glycoprotein; R-CHOP—rituximab, cyclophosphamide, doxorubicin, vincristine, and prednisone; RU486—mifepristone/glucocorticoid receptor antagonist; siRNA—small interfering RNA.

Reference	Study Type	Aim	Design/Model	Number of Subjects/Samples	Main Quantitative/Qualitative Findings	Conclusions
Cufer et al., 2000 [34]	Ex vivo study	To investigate P-gp-mediated protection against xenobiotic-induced adrenocortical injury and the effects of P-gp blockade on mitotane- and DOX-induced adrenotoxicity	Ex vivo perfused bovine adrenal glands; valspodar, mitotane, DOX, and combination treatments; assessment of cortisol secretion and xenobiotic-induced adrenocortical damage	Perfused bovine adrenal glands: control (*n* = 6), valspodar (*n* = 6), mitotane (*n* = 6), mitotane + valspodar (*n* = 6), DOX (*n* =11), and DOX + valspodar (*n* = 11)	Enhanced mitotane/DOX-induced cortisol suppression and adrenocortical damage following P-gp inhibition, particularly in the zona fasciculata	Protective role of P-gp in the adrenal cortex; P-gp blockade enhances mitotane- and DOX-induced adrenocortical damage
Owattanapanich et al., 2018 [37]	Clinical study	To evaluate the incidence and duration of adrenal insufficiency and HPA-axis suppression during R-CHOP/CHOP treatment	Prospective clinical study; newly diagnosed DLBCL patients receiving 6–8 R-CHOP/CHOP cycles; adrenal function and HPA-axis recovery assessed by serial 1-μg ACTH stimulation tests	DLBCL patients (*n* = 10): R-CHOP (*n* = 8), CHOP (*n* = 2)	Baseline adrenal function was normal in all patients. Adrenal insufficiency occurred in 30% of patients (first detected after cycle 3, peak after cycle 5). HPA-axis recovery within 3–5 weeks in most patients; persistent suppression ≥ 90 days in one patient	R-CHOP/CHOP-associated transient adrenal insufficiency and HPA-axis suppression in DLBCL patients; independent effect of DOX could not be established due to concomitant corticosteroid and multidrug treatment
Robison et al., 1985 [100]	In vivo study	To study acute changes in plasma cortisol, blood chemistry, blood pressure, and heart rate following intravenous DOX infusion	In vivo rabbit model; intravenous DOX infusion; plasma cortisol, blood chemistry, blood pressure, and heart rate assessment	Rabbits (*n* = 8): DOX (*n* = 4), control (*n* = 4)	Increased plasma cortisol and creatinine levels and decreased plasma insulin, blood pressure, and heart rate after intravenous DOX infusion	Endocrine, metabolic, and cardiovascular alterations following DOX infusion; cortisol elevation likely secondary to systemic effects rather than direct adrenal stimulation
Yang et al., 2018 [101]	In vitro and ex vivo study	To determine tissue distribution and activity of human 11β-HSD1 and its contribution to DOX-to-DOXOL conversion	In vitro/ex vivo study; human tissue fractions, hepatocytes, recombinant 11β-HSD1; assessment of 11β-HSD1 tissue distribution/activity, DOX metabolism, and contribution to DOXOL formation	Human liver microsomes (*n* = 31); pooled human liver microsomes (*n* = 50); human liver cytosol (*n* = 16); cryopreserved human hepatocytes (*n* = 10). DOX metabolism contribution assays (*n* = 5 independent experiments)	Predominant 11β-HSD1 expression in liver microsomes; DOX-to-DOXOL conversion and substantial contribution to DOXOL formation in hepatocytes, supporting a role in DOX metabolism	11β-HSD1-mediated DOXOL formation; important pathway of hepatic DOX metabolism
Kassner et al., 2008 [102]	In vitro study	To identify enzymes responsible for DOX-to-DOXOL conversion in human tissues and interindividual variability in their activity and expression	In vitro study in human liver microsomes, liver cytosol, hepatocytes, recombinant reductases, and tissue cytosols; assessment of DOX-to-DOXOL reduction, enzyme kinetics, inhibitor sensitivity, and interindividual variability in CBR1 expression and activity	Human tissue cytosols: liver (*n* = 6), stomach (*n* = 6), colon (*n* = 4), kidney (*n* = 5), heart (*n* = 10), skeletal muscle (*n* = 2), lung (*n* = 4); liver cytosols (*n* = 80); DNA samples (*n* = 57)	CBR1 and AKR1C3 as major DOX-reducing enzymes; CBR1 as the predominant hepatic DOX reductase; >70-fold variability in CBR1 expression and >22-fold variability in DOX-reducing activity; no association between DOX-reducing activity and CBR1 gene variants	Predominant role of CBR1 in hepatic DOX-to-DOXOL conversion; interindividual variability in CBR1 expression and activity may influence DOX metabolism, treatment response, and cardiotoxicity
Narang et al., 2008 [103]	In vitro and vivo study	To investigate the effects of dexamethasone on multidrug resistance transporter expression and functional activity at the rat blood–brain barrier	In vitro/in vivo dexamethasone-treated rat models; multidrug resistance transporter expression/activity, glucocorticoid receptor involvement, and PXR expression assessment	Primary rat brain microvascular endothelial cells (≥3 independent experiments); rats (*n* = 20): 4 treatment groups (*n* = 5/group)	Increased BCRP, P-gp, and MRP2 expression and reversible efflux activity after dexamethasone exposure; partial RU486-sensitive regulation of BCRP and P-gp, but not MRP2, indicating differential glucocorticoid receptor involvement	Dexamethasone-mediated regulation of multidrug resistance transporters at the blood–brain barrier; role of glucocorticoid signaling in transporter-mediated drug efflux
Michas et al., 2011 [105]	In vitro and ex vivo study	To determine the effects of dexamethasone on 11β-HSD1 expression/activity and the role of 11β-HSD1 in glucocorticoid-induced inhibition of smooth muscle cell proliferation	In vitro/ex vivo study; human coronary artery smooth muscle cells and murine aortic rings; dexamethasone treatment and siRNA-mediated downregulation of 11β-HSD1, GR, C/EBPβ, or C/EBPδ; assessment of 11β-HSD1 expression/activity, glucocorticoid signaling, and cell proliferation	HCASMC (≥3 independent experiments); proliferation assays (*n* = 4); murine aortic rings from mice (*n* = 12)	GR- and C/EBPβ/C/EBPδ-dependent upregulation of 11β-HSD1 by dexamethasone; increased smooth muscle cell proliferation and reduced dexamethasone antiproliferative effects following 11β-HSD1 downregulation	11β-HSD1-mediated glucocorticoid signaling; enhanced local glucocorticoid activation and reduced vascular smooth muscle cell proliferation
Morgan et al., 2017 [107]	In vitro, ex vivo and in vivo study	To evaluate the role of CBR1 in cortisol metabolism, GR signaling, and obesity-related metabolic complications	In vitro/ex vivo/in vivo study; enzymatic and cell-based GR activation assays, human, equine, and murine tissues, and a diet-induced obesity mouse model; assessment of CBR1-dependent glucocorticoid metabolism, GR signaling, and metabolic effects of CBR1 inhibition	Horses: urine, plasma, and adipose tissue samples from lean (*n* = 14) and obese (*n* = 14) animals; humans: urine samples [lean (*n* = 15), obese (*n* = 37)], plasma samples [lean (*n* = 10), obese (*n* = 10)], adipose tissue biopsies [lean (*n* = 8), obese (*n* = 8)]; mice: vehicle (*n* = 12) and quercetin (*n* = 12) groups, with adipose tissue and liver analyses	CBR1-mediated conversion of cortisol to 20β-DHF (weak endogenous GR agonist); increased CBR1 expression and 20β-DHF production in obesity; CBR1 inhibition impaired glucose tolerance and enhanced hepatic GR signaling, suggesting protection against excessive GR activation	CBR1-mediated modulation of glucocorticoid metabolism and GR activation; CBR1–20β-dihydrocortisol pathway as a novel link between glucocorticoid biology and obesity-associated metabolic dysfunction

**Table 2:** Summary of studies investigating glucocorticoid-related mechanisms and pathways relevant to DOX-induced cardiotoxicity. Abbreviations: 11β-HSD—11β-hydroxysteroid dehydrogenase; 11β-HSD2—11β-hydroxysteroid dehydrogenase type 2; Ca^2+^—calcium ions; CV1—African green monkey kidney fibroblast-like cells; DOX—doxorubicin; ELL—nineteen lysine-rich leukemia; eNOS—endothelial nitric oxide synthase; GR—glucocorticoid receptor; HF—heart failure; HIF1/HIF1α—hypoxia-inducible factor 1/hypoxia-inducible factor 1 alpha; L-NAME—Nω-nitro-L-arginine methyl ester; MR—mineralocorticoid receptor; NO—nitric oxide; NOx—nitrate, nitrite, and nitric oxide; p22phox—essential NADPH oxidase subunit encoded by CYBA; PASMC—pulmonary artery smooth muscle cells; p-eNOS—phosphorylated eNOS; ROS—reactive oxygen species; Top2β—topoisomerase IIβ.

Reference	Study Type	Aim	Design/Model	Number of Subjects/Samples	Main Quantitative/Qualitative Findings	Conclusions
Zhang et al., 2012 [22]	In vivo study	To investigate the role of cardiomyocyte Top2β in DOX-induced cardiotoxicity and the mechanisms underlying DOX-induced mitochondrial dysfunction and cardiac injury	In vivo/ex vivo cardiomyocyte-specific Top2β knockout and wild-type mice; acute/chronic DOX treatment; assessment of DNA damage, apoptosis, transcriptomic alterations, mitochondrial biogenesis, oxidative stress, and cardiac function	Mice: acute DNA damage/apoptosis analyses (*n* = 4/group); gene expression and mitochondrial function analyses (*n* = 3/group); chronic cardiac assessment (*n* = 7/group)	Top2β-dependent DOX-induced DNA damage, cardiomyocyte apoptosis, mitochondrial dysfunction, and ROS generation; attenuation of these effects by cardiomyocyte-specific Top2β deletion; protection against chronic DOX-induced LV dysfunction in Top2β-deficient mice	Top2β-dependent DNA damage as a key mediator of DOX-induced transcriptional and mitochondrial alterations, oxidative stress, apoptosis, and progressive cardiac dysfunction
He et al., 2019 [120]	In vitro and in vivo study	To investigate the role of mitochondrial ROS generation, eNOS/NO pathway impairment, and mitochondrial dysfunction in DOX-induced endothelial injury	In vivo/in vitro DOX-treated C57BL/6J mice and HUVECs; assessment of endothelial injury, vascular function, apoptosis, ROS generation, eNOS/NO signaling, and mitochondrial function	Mice (*n* = 15/group; total *n* = 60); HUVECs (*n* = 8 independent experiments)	DOX-induced endothelial injury with impaired vascular function, reduced eNOS/p-eNOS expression and NO bioavailability, increased apoptosis, ROS generation, and mitochondrial dysfunction; DOX-induced mitochondrial ROS generation preceding cytoplasmic ROS production; protective effects of edaravone, cyclosporin A, and eNOS overexpression; exacerbation of DOX-induced endothelial injury by L-NAME	Mitochondrial ROS generation, eNOS/NO pathway impairment, and mitochondrial dysfunction are key mediators of DOX-induced endothelial injury and vascular dysfunction
Segar et al., 1995 [26]	In vivo study	To determine the effects of cortisol on fetal RAS gene expression	In vivo fetal sheep exposed to cortisol or saline for 48 h; assessment of RAS gene expression in kidney, liver, adrenal, and cardiac tissues	Twin fetal sheep pairs (*n* = 10); one cortisol-treated fetus and one co-twin control per pair	Tissue-specific cortisol-induced regulation of RAS gene expression; increased cardiac AT1 receptor expression; decreased renal and hepatic AT1 receptor expression; reduced renal renin and hepatic angiotensinogen expression	Tissue-specific cortisol-dependent regulation of fetal RAS gene expression; increased cardiac AT1 receptor expression; reduced renal and hepatic RAS components
Omori et al., 2014 [108]	In vivo and in vitro study	To investigate the role of ELL in MR-mediated glucocorticoid-induced cardiac fibrosis under oxidative stress	In vivo/in vitro Dahl salt-sensitive rats with heart failure and neonatal rat cardiac fibroblasts; assessment of cardiac fibrosis, oxidative stress, ELL/MR signaling, and collagen synthesis	In vivo: HF and control rats (*n* = 8/group; ELL expression analysis, *n* = 5/group). In vitro: ELL/MR/11β-HSD2 expression analyses (*n* = 4–5/group); ELL knockdown verification (*n* = 3/group); collagen synthesis assays (*n* = 8/group)	Cardiac fibrosis in HF associated with increased oxidative stress and ELL–MR signaling; oxidative stress-enhanced ELL–MR interaction without altered MR expression; MR-dependent corticosterone-induced collagen synthesis	Oxidative stress-dependent MR activation and profibrotic signaling; ELL as a key mediator of MR-dependent collagen synthesis and cardiac fibrosis
Rogers et al., 2002 [74]	In vitro study	To investigate mechanisms underlying cortisol-induced suppression of NO production and eNOS expression in coronary artery endothelial cells	In vitro cortisol-treated bovine coronary artery endothelial cells; assessment of NO production, eNOS expression, GR signaling, and intracellular Ca^2+^ mobilization	NOx production analyses (*n* = 4–5); GR analysis (*n* = 3); eNOS expression and degradation analyses (*n* = 5); intracellular Ca^2+^ mobilization analysis (*n* = 5)	Cortisol-induced suppression of NO production, reduced eNOS expression, accelerated eNOS degradation, and impaired intracellular Ca^2+^ mobilization; GR- and 11β-HSD-dependent effects	Cortisol-induced impairment of coronary endothelial NO signaling via reduced eNOS expression, accelerated eNOS degradation, and impaired Ca^2+^-dependent NO production; 11β-HSD-dependent regulation of endothelial function
Kračun et al., 2020 [117]	In vitro, ex vivo, and in vivo study	To investigate the role of NADPH oxidases and HIF1 signaling in glucocorticoid-induced ROS generation and cardiovascular dysfunction	In vitro/ex vivo/in vivo dexamethasone-treated human microvascular endothelial cells, PASMC, murine vascular tissues, and mouse models of glucocorticoid excess; assessment of ROS generation, HIF1 signaling, angiogenic responses, pulmonary vascular remodeling, and cardiac function	In vitro ROS and signaling analyses in HMEC-1 and PASMC (*n* = 4); ex vivo vascular tissue analyses (*n* = 4); in vivo cardiac and pulmonary analyses (*n* = 3/group, depending on the assay)	Glucocorticoid-induced ROS generation via p22phox-, NOX2-, and NOX4-dependent NADPH oxidase signaling; p22phox-dependent HIF1 activation and angiogenic responses; 11β-HSD2 deficiency-induced pulmonary vascular remodeling, pulmonary hypertension, and cardiac dysfunction attenuated by p22phox disruption	NADPH oxidase-dependent ROS generation and HIF1 activation as key mediators of glucocorticoid-induced pulmonary vascular remodeling, pulmonary hypertension, and cardiac dysfunction
Ali et al., 2021 [116]	In vitro study	To investigate the role of physiological 11β-HSD2 expression in regulating corticosterone-induced MR activation and nuclear translocation	In vitro MR-expressing Chinese hamster ovary and CV1 cells with varying 11β-HSD2 expression; assessment of MR transactivation, corticosterone metabolism, and MR nuclear translocation	MR transactivation and nuclear translocation analyses (*n* = 3); corticosterone metabolism analyses (*n* = 4); MR protein analyses (*n* = 3)	Similar aldosterone- and corticosterone-induced MR activation in the absence of 11β-HSD2; 11β-HSD2-dependent attenuation of corticosterone-induced MR transactivation and nuclear translocation; dose-dependent and corticosterone-selective effects	11β-HSD2-mediated protection of MR from corticosterone-induced activation and preservation of aldosterone selectivity
Huang et al., 2013 [125]	In vivo study	To investigate the impact of glucocorticoid-induced suppression of early inflammatory responses on cardiac repair following myocardial injury	In vivo zebrafish cardiac injury model treated with beclomethasone or vehicle; assessment of cardiac repair, inflammatory responses, phagocyte recruitment, angiogenesis, collagen deposition, and cell proliferation	MR transactivation and nuclear translocation analyses (*n* = 3); corticosterone metabolism analyses (*n* = 4); MR protein analyses (*n* = 3)	Glucocorticoid-induced impairment of cardiac repair with increased scar tissue and collagen deposition; suppression of early inflammatory responses and phagocyte recruitment; reduced angiogenesis and cell proliferation	Glucocorticoid-induced early inflammatory response suppression and impaired cardiac regeneration

**Table 3:** Summary of experimental studies on apelin/APJ signaling, HPA-axis activation, and cortisol-related mechanisms relevant to DOX-induced cardiotoxicity. Abbreviations: ACTH—adrenocorticotropic hormone; APJ—apelin receptor; APJ KO—APJ knockout; APLN—apelin; AVP—arginine vasopressin; CRF—corticotropin-releasing factor; CRH—corticotropin-releasing hormone; DOX—doxorubicin; ECG—electrocardiography; ELA—elabela; HPA axis—hypothalamic–pituitary–adrenal axis; L-NAME—Nω-nitro-L-arginine methyl ester; LPS—lipopolysaccharide; miR-631—microRNA-631; mRNA—messenger RNA; NPY—neuropeptide Y; PVN—paraventricular nucleus; QTc—corrected QT interval; RNA-seq—RNA sequencing; V1bR—vasopressin V1b receptor.

Reference	Study Type	Aim	Design/Model	Number of Subjects/Samples	Main Quantitative/Qualitative Findings	Conclusions
Hamada et al., 2015 [43]	In vitro and in vivo study	To investigate the role of the apelin–APJ system in DOX-induced cardiotoxicity and the cardioprotective effects of apelin–APJ activation	In vivo/in vitro models: DOX-treated mice and APJ-expressing H9c2 cardiomyoblasts; assessment of apelin–APJ system expression, cardiac injury, and apelin/APJ-mediated cytoprotection	Mice (*n* = 3–12/group, depending on assay); survival analysis: wild-type (*n* = 41), APJ heterozygous (*n* = 41), APJ knockout (*n* = 12); APJ-expressing H9c2 cardiomyoblasts	DOX-induced downregulation of cardiac apelin–APJ signaling; exacerbation of cardiac dysfunction, mortality, oxidative protein damage, and autophagic impairment in APJ-deficient models; protection against DOX-induced cardiomyoblast cytotoxicity by APJ overexpression and apelin treatment	Endogenous cardioprotective role of the apelin–APJ pathway in DOX-induced cardiotoxicity; exacerbation of cardiac dysfunction, oxidative damage, and autophagic impairment following APJ signaling loss
Buczma et al., 2025 [140]	In vivo study	To investigate the role of the apelinergic system in DOX-induced electrocardiographic abnormalities and left ventricular systolic dysfunction	Sprague Dawley rats with chronic DOX-induced cardiotoxicity; apelin-13, ELA, or ML221 treatment; assessment of electrocardiographic abnormalities and left ventricular systolic function by ECG and transthoracic echocardiography	Rats (*n* = 54): control, DOX, APLN 40, APLN 200, ELA 40, ELA 200, and ML221 groups; group sizes not specified	DOX-induced QT/QTc prolongation and left ventricular systolic dysfunction; attenuation of electrocardiographic abnormalities and preservation of systolic function by low-dose APLN-13 and ELA; lack of protection with higher doses and APJ antagonism	Apelinergic system as a key modulator of DOX-induced cardiac electrical and systolic dysfunction; cardioprotective effects of moderate APJ activation, but not excessive APJ stimulation or APJ antagonism
Chen et al., 2023 [141]	In vitro study	To investigate the role of APLN in DOX resistance and the effects of miR-631 restoration on DOX sensitivity in chondrosarcoma cells	In vitro DOX-resistant and parental chondrosarcoma cell lines (SW1353 and JJ012); assessment of APLN expression, miR-631-mediated APLN regulation, and effects on DOX resistance	W1353/SW1353R RNA-seq and cell death assays (*n* = 3 experiments); chondrosarcoma tissue microarray: low-grade (*n* = 13), high-grade (*n* = 39); SW1353R/JJ012R functional assays: *n* not specified	Increased APLN expression and signaling associated with DOX resistance in chondrosarcoma cells; enhanced DOX sensitivity and apoptosis following APLN knockdown; reduced APLN expression and restored DOX sensitivity following miR-631 overexpression; higher APLN expression in high-grade chondrosarcoma tissues	miR-631/APLN pathway as a key regulator of DOX resistance in chondrosarcoma cells; APLN-mediated chemoresistance and restoration of DOX sensitivity through miR-631-mediated APLN suppression
Newson et al., 2009 [143]	In vivo study	To investigate the role of central apelin in HPA-axis activation and the involvement of CRF- and AVP-dependent mechanisms	In vivo wild-type and V1bR knockout mice; intracerebroventricular pGlu-apelin-13 ± CRF receptor antagonism; assessment of HPA-axis activation by plasma ACTH and corticosterone	CRF antagonist groups (*n* = 12–16/group); V1bR knockout groups (*n* = 9–12/group); V1bR knockout + CRF antagonist groups (*n* = 12–16/group); hormone assays in duplicate; experiments performed ≥2 times	Central pGlu-apelin-13-induced increases in plasma ACTH and corticosterone; attenuation by CRF receptor blockade and V1bR deletion; contribution of CRF- and AVP-dependent pathways to apelin-induced HPA-axis activation	Central apelin as a key regulator of HPA-axis activation; CRF- and AVP-dependent mediation of apelin-induced ACTH and corticosterone secretion
Reaux-Le Goazigo et al., 2007 [145]	Ex vivo study	To investigate the expression of the apelin/APJ system in anterior pituitary corticotrophs and the effects of apelin on ACTH release	Ex vivo adult male rat anterior pituitaries; assessment of apelin/APJ localization and apelin-induced ACTH secretion by tissue localization analyses and pituitary perifusion	Apelin/APJ localization: *n* not specified (12 images/pituitary); perifusion experiments: *n* = 3–5 chambers/condition (basal ACTH) and *n* = 3 chambers/condition (K^+^-evoked ACTH); 1 anterior pituitary/chamber	High apelin/APJ expression in anterior pituitary corticotrophs; active apelin-induced stimulation of basal and K^+^-evoked ACTH secretion ex vivo; inactive apelin fragment ineffective	Pituitary apelinergic system as a key regulator of ACTH secretion; direct stimulation of corticotroph activity by apelin through autocrine/paracrine mechanisms
Charles et al., 2006 [147]	In vivo study	To investigate the integrated hemodynamic, hormonal, and renal effects of apelin-13 in a large conscious animal model	In vivo conscious sheep; incremental intravenous apelin-13 administration; assessment of hemodynamic, ECG, endocrine, natriuretic peptide, cyclic nucleotide, and renal responses	Conscious sheep (*n* = 10); crossover vehicle/apelin-13 treatment	High-dose apelin-13-induced hemodynamic, neuroendocrine, and ECG changes; increased AVP, ACTH, aldosterone, cortisol, natriuretic peptides, and cyclic nucleotides; no significant effects on renal indices	Key role of apelin in cardiovascular and neuroendocrine homeostasis; regulation of hemodynamic responses, HPA-axis activity, and natriuretic peptide release
Taheri et al., 2002 [149]	In vivo and ex vivo study	To investigate the role of central apelin-13 in the regulation of water intake, HPA-axis activity, and hypothalamic CRH/AVP release	In vivo rats/ex vivo rat hypothalamic explants; intracerebroventricular apelin-13 administration; assessment of food and water intake, pituitary hormone secretion, and hypothalamic neuropeptide release	In vivo rats (*n* = 7–10/group); ex vivo hypothalamic explants (*n* = 20–30/group)	Dose-dependent increase in water intake; increased plasma ACTH and corticosterone with reduced prolactin, LH, and FSH; increased hypothalamic CRH and AVP release ex vivo; no effect on NPY release	Key role of apelin in hypothalamic regulation of water intake and neuroendocrine activity; contribution of CRH- and AVP-dependent pathways to HPA-axis regulation
O’Carroll et al., 2003 [151]	In vivo study	To investigate the regulation of hypothalamic APJ receptor mRNA expression by acute and repeated stress and endogenous glucocorticoids	In vivo male Wistar rats; acute/repeated restraint stress; assessment of PVN APJ receptor mRNA expression and glucocorticoid-mediated APJ regulation	Rats (*n* = 3–4/group); brain sections (*n* = 4–6/rat)	Acute- and repeated stress-induced increases in parvocellular PVN APJ receptor mRNA expression (greater after acute stress); marked upregulation after adrenalectomy; no further stress-induced increase in adrenalectomized rats; no significant changes in the magnocellular PVN	Stress- and glucocorticoid-dependent regulation of APJ receptor expression in the hypothalamic PVN; involvement of the apelin/APJ system in neuroendocrine stress responses
Jászberényi et al., 2004 [152]	In vivo study	To investigate the behavioral, endocrine, and thermoregulatory effects of centrally administered apelin-13 and the involvement of CRH-mediated HPA-axis activation	In vivo rats; intracerebroventricular apelin-13 administration; assessment of HPA-axis activation, behavioral and thermoregulatory responses, and mechanisms mediating apelin-13 actions	Rats: control (*n* = 8); apelin-13 dose-response groups (*n* = 6/group); CRH antagonist groups (*n* = 6/group); haloperidol groups (*n* = 6–8/group); L-NAME groups (*n* = 5–7/group)	Apelin-13-induced increases in plasma corticosterone and HPA-axis activation in vivo; CRH-dependent corticosterone response; dopamine-, NO-, and prostaglandin-related mediation of behavioral and thermoregulatory effects	CRH-dependent activation of the HPA axis and corticosterone release by central apelin-13; role of apelin in neuroendocrine stress regulation
Newson et al., 2013 [155]	In vivo study	To investigate the role of APJ in HPA-axis responses to acute stressors and the stressor- and sex-dependent regulation of APJ expression	In vivo APJ KO and wild-type mice exposed to acute stressors; assessment of HPA-axis responses and stressor- and sex-dependent APJ regulation	Mice: basal CORT rhythm (*n* = 3–6/time point); mild restraint (*n* = 5–7/group); LPS challenge (*n* = 4–5/group); insulin-induced hypoglycemia (*n* = 5–6/group); forced swim (*n* = 4–7/group)	Normal basal HPA-axis function and neuroendocrine morphology in APJ KO mice; attenuated HPA-axis responses to LPS, insulin-induced hypoglycemia, and forced swim stress; preserved responses to mild restraint; stressor-dependent role of APJ in neuroendocrine stress regulation	APJ as a physiological modulator of HPA-axis stress responses; stressor-specific and partially sex-dependent regulation of neuroendocrine activity

## Data Availability

No new data were created or analyzed in this study.

## Abbreviations

The following abbreviations are used in this manuscript:

11β-HSD1	11β-hydroxysteroid dehydrogenase 1
11β-HSD2	11β-hydroxysteroid dehydrogenase 2
ABCB1	ATP-binding cassette 1 subfamily B
ACE	angiotensin-converting enzyme
ACE2	angiotensin-converting enzyme 2
ACTH	adrenocorticotropic hormone
AGT	angiotensinogen
AMPK	AMP-activated protein kinase
ANG	angiotensinogen
APJ	apelin receptor
AT_1_	angiotensin II type 1 receptor
ATP	adenosine triphosphate
AVP	vasopressin
CBG	transcortin or corticosteroid-binding globulin
CD8^+^	cytotoxic cluster of differentiation 8
CRH	corticotropin-releasing hormone
DOX	doxorubicin
DNA	deoxyribonucleic acid
eNOS	endothelial nitric oxide synthase
GCs	glucocorticoids
GR	glucocorticoid receptor
HSP	heat shock proteins
IL-10	interleukin 10
IL-2	interleukin 2
MR	mineralocorticoid receptor
mRNA	messenger ribonucleic acid
P-gp	P-glycoprotein
PVN	paraventricular nucleus
RAAS	renin–angiotensin–aldosterone system
ROS	reactive oxygen species
StAR	steroidogenic acute regulatory protein
Th1	T helper 1 cells
